# Nomogram to predict live birth in infertile women with adenomyosis undergoing IVF/ICSI treatment: a retrospective cohort study

**DOI:** 10.3389/fendo.2025.1473923

**Published:** 2025-07-17

**Authors:** Zhongyuan Li, Wei Chen, Xiaohan Sun, Xueqing Zhao, Jingmei Hu, Li Ge, Wenting Wang, Ping Zhang

**Affiliations:** ^1^ Center for Reproductive Medicine, The Second Hospital of Shandong University, Cheeloo College of Medicine, Shandong University, Jinan, Shandong, China; ^2^ School of Radiology, Shandong First Medical University and Shandong Academy of Medical Sciences, Tai’an, Shandong, China; ^3^ Institute of Women, Children and Reproductive Health, Shandong University, Jinan, Shandong, China; ^4^ State Key Laboratory of Reproductive Medicine and Offspring Health, Shandong University, Jinan, Shandong, China; ^5^ National Research Center for Assisted Reproductive Technology and Reproductive Genetics, Shandong University, Jinan, Shandong, China; ^6^ Key laboratory of Reproductive Endocrinology (Shandong University), Ministry of Education, Jinan, Shandong, China; ^7^ Shandong Technology Innovation Center for Reproductive Health, Jinan, Shandong, China; ^8^ Shandong Provincial Clinical Research Center for Reproductive Health, Jinan, Shandong, China; ^9^ Shandong Key Laboratory of Reproductive Medicine, Shandong Provincial Hospital Affiliated to Shandong First Medical University, Jinan, Shandong, China; ^10^ Research Unit of Gametogenesis and Health of Assisted Reproductive Technology (ART)-Offspring, Chinese Academy of Medical Sciences (No.2021RU001), Jinan, Shandong, China; ^11^ Center for Reproductive Medicine, Cheeloo College of Medicine, Shandong University, Jinan, Shandong, China; ^12^ Gynecologic Department, The Second Hospital of Shandong University, Cheeloo College of Medicine, Shandong University, Jinan, Shandong, China

**Keywords:** adenomyosis, live birth, nomogram, *in vitro* fertilization (IVF), intracytoplasmic sperm injection (ICSI)

## Abstract

**Purpose:**

The study aimed to develop a nomogram to predict live birth outcomes in infertile women with adenomyosis undergoing IVF/ICSI treatment.

**Materials and methods:**

Data were collected from the Center for Reproductive Medicine, Shandong University, between January 2016 and December 2020. This study included 222 fresh embryo transfer (ET) cycles and 266 frozen embryo transfer (FET) cycles. Patients were divided into a modeling cohort and a validation cohort. In the modeling cohort, multivariable logistic regression was used to generate the predictive model and construct the nomogram. The predictive model was subsequently validated and calibrated.

**Results:**

In total, 249 patients (51.0%) achieved clinical pregnancy, and 165 patients (33.8%) had a live birth. Predictive variables in the final model included mean initial uterine diameter, age, body mass index (BMI), type of infertility (primary or secondary), stage of transferred embryo, endometrial thickness, number of embryos transferred, and pregnancy type. The area under the receiver operating characteristic (ROC) curve for the final prediction model was 0.915 (95% confidence interval [CI]: 88.6%–94.3%) in the modeling cohort and 0.940 (95% CI: 90.3%–97.6%) in the validation cohort. Calibration curves and the Hosmer–Lemeshow (H–L) test demonstrated excellent consistency between predicted and actual live birth outcomes.

**Conclusions:**

This study established a well-calibrated predictive model capable of accurately forecasting live birth outcomes in infertile women with adenomyosis undergoing IVF/ICSI treatment.

## 1 Introduction

Adenomyosis significantly affects women’s quality of life and is characterized by an invasion of the myometrium by endometrial glands and stroma (1). It commonly manifests as pelvic pain, excessive uterine bleeding, anemia, and infertility (2–4). While approximately one-third of cases are asymptomatic, the symptoms that do occur are often nonspecific (5). Historically, adenomyosis was believed to primarily impact older women, and earlier studies reported no statistically significant differences in pregnancy outcomes between women with and without the disease. However, recent research has demonstrated an increasing prevalence of adenomyosis in younger women, indicating its detrimental impact on fertility (6). Additionally, studies have shown that adenomyosis increases the risk of several adverse obstetric outcomes, such as preeclampsia, preterm delivery, fetal malpresentation, postpartum hemorrhage, low birth weight, and small-for-gestational-age infants (7–9).

Among women undergoing IVF/ICSI treatment for infertility, adenomyosis affects approximately 30%-40% of patients (10). Assisted reproductive technology (ART) remains a crucial treatment option for infertility associated with adenomyosis (11, 12). However, multiple studies have reported reduced embryo implantation rates, clinical pregnancy rates, ongoing pregnancy rates, and live birth rates in women with adenomyosis compared to those without Furthermore, women with adenomyosis have an increased risk of miscarriage (10, 13). The probability of achieving a live birth using ART in patients with adenomyosis-associated infertility depends on various factors. Current guidelines recommend several scoring systems for evaluating pregnancy outcomes in adenomyosis patients undergoing IVF/ICSI treatments. However, these scoring systems are population-based rather than individualized.

In ART, the ability to accurately predict live birth outcomes following IVF/ICSI is critical for both clinical decision-making and patient counseling. A nomogram, based on multivariate regression analysis, integrates multiple key predictors by assigning scores to each factor according to its contribution (as indicated by regression coefficients) and combining these scores to predict outcomes visually. Nomograms translate complex regression models into simple, intuitive graphical representations, thus facilitating patient evaluation. Because of their user-friendly format and interpretability, nomograms are increasingly used in medical research and clinical practice, supporting evidence-based, individualized treatment plans and effective communication between clinicians and patients. Some studies have developed predictive models specifically for live birth outcomes following frozen embryo transfer (FET) in patients with adenomyosis (14). However, few studies have evaluated live birth rates following both fresh embryo transfer (ET) and frozen embryo transfer (FET) in infertile women with adenomyosis.

Therefore, this study aimed to develop a nomogram based on retrospective data analysis, providing clinicians with a reliable reference tool for predicting the probability of live birth in patients with adenomyosis undergoing IVF/ICSI treatment.

## 2 Materials and methods

### 2.1 Study design and population

Baseline data were extracted from the ART electronic medical system. A retrospective analysis was performed on women diagnosed with adenomyosis who underwent their first IVF/ICSI cycle at the Center for Reproductive Medicine, Shandong University, between January 2016 and December 2020.

The data screening process comprised four steps: 1. Preliminary data extraction: Medical records of all women who had undergone IVF/ICSI treatment within the past 5 years were retrieved from the hospital’s electronic medical record system. Data collected included age, IVF cycle indicators, ultrasound reports, blood tests, and embryonic laboratory records. 2. Confirmation of adenomyosis: Diagnosis was established based on the Morphological Uterus Sonographic Assessment (MUSA) group consensus from the MUSA group, using ultrasound features such as a globular asymmetric uterus, heterogeneous myometrium, asymmetric thickening, hyperechoic islands, fan-shaped shadowing, irregular or interrupted junctional zone, echogenic subendometrial lines and buds, cysts, and translesional vascularity (15, 16). To ensure diagnostic consistency, all transvaginal ultrasounds were conducted by at least two experienced sonographers, each with over 8 years of experience in gynecologic imaging. 3. Inclusion criteria: (i) a confirmed diagnosis of adenomyosis based on MUSA criteria; (ii) age under 45 years; (iii) undergoing a first IVF/ICSI cycle; and (iv) no evidence of uterine malformations, intrauterine lesions, or untreated hydrosalpinx. 4. Data verification and quality control: Two researchers independently verified critical data such as medication dosages and timing of embryo transfer, ensured chronological accuracy of examinations and treatments, and contacted hospital archives to supplement missing records.

This study received ethical approval from the Ethics Committee of the Center for Reproductive Medicine, Shandong University (No. 2021-133).

### 2.2 IVF/ICSI treatment procedure

Depending on sperm quality, clinicians selected either IVF or ICSI. The ovarian stimulation protocol was determined based on the patient’s specific clinical characteristics and included the GnRH-a long protocol, long protocol, short protocol, antagonist protocol, and other unconventional protocols, as described in previous studies (17).

During controlled ovarian hyperstimulation (COH), follicular development was continuously monitored by transvaginal ultrasound and serum hormone measurements. When at least two follicles reached a diameter of ≥18 mm, ovulation was triggered by intramuscular injection of 8,000–10,000 IU of HCG, followed by transvaginal oocyte retrieval 34–36 h later. In fresh ET cycles, two high-quality cleavage-stage embryos were transferred on day 3, or one high-quality blastocyst on day 5, under transvaginal ultrasound guidance (18). Luteal phase support for fresh cycles began after oocyte retrieval and consisted of vaginal progesterone soft capsules (Utrogestan^®^, Besins, Belgium, 200 mg once daily) and oral dydrogesterone (Duphaston^®^, Abbott, Netherlands, 20 mg twice daily), or vaginal progesterone gel (Crinone gel^®^, Merck Serono, Switzerland, 90 mg once daily) combined with oral dydrogesterone tablets (Duphaston^®^, Abbott, Netherlands, 10 mg twice daily) (17). Luteal phase support in fresh cycles began post-oocyte retrieval and included vaginal progesterone capsules (Utrogestan^®^, Besins, Belgium, 200 mg once daily), oral dydrogesterone (Duphaston^®^, Abbott, Netherlands, 20 mg twice daily), or vaginal progesterone gel (Crinone gel^®^, Merck Serono, Switzerland, 90 mg once daily) combined with oral dydrogesterone (Duphaston^®^, Abbott, Netherlands, 10 mg twice daily) (17). Luteal phase support lasted for approximately 12 weeks of gestation.

The “freeze-all” strategy, or cancellation of fresh ET, was applied in cases of high risk for ovarian hyperstimulation syndrome, endometrial asynchrony, or hydrosalpinx. High-quality blastocysts graded ≥4BC were vitrified for subsequent frozen cycles. Endometrial preparation protocols for FET cycles included GnRHa pretreatment and artificial cycles, natural cycles, artificial cycles, and ovulation induction cycles. In patients with severe adenomyosis, GnRHa pretreatment (3.75 mg monthly) was administered and repeated if the anteroposterior uterine diameter exceeded 70 mm, with a maximum of six injections. Artificial cycles were initiated four weeks after the final injection. Progesterone supplementation was started once endometrial thickness reached ≥0.7 cm, followed by transfer of one thawed blastocyst five days later under ultrasound guidance. Additional FET preparation protocols have been described elsewhere (19).

Live birth was defined as the delivery of an infant exhibiting spontaneous breathing or other signs of life after 28 weeks of gestation.

### 2.3 Data analysis

All statistical analyses were conducted using the RMS package in R (version 4.3.0) and SPSS 27.0 software. Participants were randomly assigned into validation (n = 147) and modeling cohorts (n = 341) using R. Continuous variables with normal distribution were expressed as mean ± standard deviation (SD), and categorical variables were presented as frequencies. Group differences were assessed using Student’s t-test or the chi-square test. A two-sided *P*-value < 0.05 was considered statistically significant.

### 2.4 Development and evaluation of the model

Multivariable logistic regression (MLR) was applied to assess associations between patient characteristics and live birth outcomes, enabling the development of the prediction model and construction of the nomogram. Variables identified as significant in univariate logistic regression analysis (*P* < 0.1), along with clinically relevant variables, were included in the multivariable model. Independent covariates were selected using backward elimination. Each variable was assigned a weighted score, and the cumulative score was used to estimate the probability of live birth.

The predictive performance of the nomogram was evaluated in the validation cohort through discrimination and calibration analyses (20). Discrimination was quantified using receiver operating characteristic (ROC) curves and the area under the curve (AUC). Calibration was assessed using the Hosmer–Lemeshow (H–L) test.

## 3 Results

### 3.1 General characteristics of infertile women with adenomyosis

Data were collected from the Center for Reproductive Medicine, Shandong University, from January 2016 to December 2020. A total of 488 embryo transfer cycles were included, comprising 222 fresh embryo transfer (ET) cycles and 266 frozen embryo transfer (FET) cycles. Patients were randomly allocated into a validation cohort (n = 147) and a modeling cohort (n = 341).

Variables considered relevant to live birth prediction, based on prior literature and clinical experience, included patient age, body mass index (BMI), duration and type of infertility, history of dysmenorrhea, presence of endometriosis, mean initial uterine diameter (calculated as the average of longitudinal width and length measured by transvaginal sonography on days 2–6 of the menstrual cycle), basal follicle-stimulating hormone (FSH), antral follicle count (AFC), anti-Müllerian hormone (AMH), type of embryo transfer (ET or FET), ovarian stimulation and FET protocols, endometrial thickness, embryo transfer stage, number of embryos transferred, and pregnancy type. These baseline characteristics are summarized in **Table 1**.

**Table 1:** General characteristics in the training and the validation cohorts.

Characteristic	Total (n = 488)	Training cohort (n = 341)	Validation cohort (n = 147)
Age (years)	33.93 ± 4.54	33.87 ± 4.44	34.08 ± 4.79
BMI (kg/m^2^)	24.27 ± 3.65	24.37 ± 3.54	24.03 ± 3.90
Duration of infertility (years)	3.45 ± 2.51	3.38 ± 2.39	3.60 ± 2.77
Primary infertility; n (%)	188 (38.5)	134 (39.3)	54 (36.7)
History of dysmenorrhea; n (%)			
None	110 (22.5)	75 (22.0)	35 (23.8)
Mild	152 (31.1)	103 (30.2)	49 (33.3)
Moderate	105 (21.5)	76 (22.3)	29 (19.7)
Severe	121 (24.8)	87 (25.5)	34 (23.1)
Endometriosis; n (%)	107 (21.9)	74 (21.7)	33 (22.4)
Mean initial uterine diameter (cm)	6.02 ± 1.42	6.01 ± 1.41	6.04 ± 1.43
Basal FSH (IU/L)	6.88 ± 2.32	6.88 ± 2.32	6.90 ± 2.31
AMH (ng/ml)	1.78 ± 0.41	1.77 ± 0.42	1.80 ± 0.40
AFC	13.36 ± 7.46	13.28 ± 7.79	13.55 ± 6.66
ET or FET; n (%)			
ET	222 (45.5)	166 (48.7)	56 (38.1)
FET	266 (54.5)	175 (51.3)	91 (61.9)
Protocol of ovarian stimulation or FET; n (%)			
GnRH-a long protocol	91 (18,6)	77 (22.6)	14 (9.5)
Other ovarian stimulation protocol	131 (26.8)	89 (26.1)	42 (28.6)
GnRHa-HRT protocol of FET	128 (26.2)	82 (24.0)	46 (31.3)
Other protocol of FET	138 (28.3)	93 (27.3)	45 (30.6)
Endometrial thickness (mm)	0.98 ± 0.23	0.98 ± 0.23	0.96 ± 0.22
Stage of embryo transfer n (%)			
Cleavage	158 (32.4)	122 (35.8)	36 (24.5)
Blastocyst	330 (67.6)	219 (64.2)	111 (75.5)
Number of embryo transfer; n (%)			
1	358 (73.4)	241 (70.7)	117 (79.6)
2	130 (26.6)	100 (29.3)	30 (20.4)
Pregnancy type; n (%)			
No pregnancy	241 (49.4)	161 (47.2)	80 (54.4)
Singleton pregnancy	199 (40.8)	141 (41.3)	58 (39.5)
Twin pregnancy	48 (9.8)	39 (11.4)	9 (6.1)

Data are expressed as mean ± SD or n (%), unless otherwise stated.

BMI, body mass index; FSH, follicle-stimulating hormone; AMH, anti-Mullerian hormone; AFC, antral follicle count; ET, embryo transfer; FET, frozen embryo transfer; GnRH, gonadotrophin-releasing hormone; HRT, hormone replacement therapy.

### 3.2 Characteristics of women in the training cohort

Patients were randomly assigned to the training cohort (n = 341) and validation cohort (n = 147). Among the 341 cycles in the training cohort, 119 (34.8%) resulted in a live birth. Women who achieved a live birth were younger (32.91 ± 3.97 years vs. 34.38 ± 4.60 years, *P* = 0.003), had a lower BMI (23.80 ± 3.29 vs. 24.67 ± 3.63 kg/m², *P* = 0.03), and smaller mean initial uterine diameter (5.71 ± 1.27 vs. 6.17 ± 1.46 cm, *P* = 0.004) compared to women who did not achieve a live birth. Protocols for ovarian stimulation, number of embryos transferred, and pregnancy type also significantly affected live birth rates, as detailed in **Table 2**.

**Table 2:** General characteristics of women in the training cohort with and without live birth.

Characteristic	With live birth (n = 119)	Without live birth (n = 222)	*P*-value
Age (years)	32.91 ± 3.97	34.38 ± 4.60	0.003*
BMI (kg/m^2^)	23.80 ± 3.29	24.67 ± 3.63	0.03*
Duration of infertility (years)	3.14 ± 2.26	3.51 ± 2.45	0.18
Primary infertility; n (%)	44 (37.0)	90 (40.5)	0.52
Degree of dysmenorrhea; n (%)			0.61
None	23 (19.3)	52 (23.4)	
Mild	38 (31.9)	65 (29.3)	
Moderate	24 (20.2)	52 (23.4)	
Severe	34 (28.6)	53 (23.9)	
Endometriosis; n (%)	31 (26.1)	43 (19.4)	0.15
Mean initial uterine diameter (cm)	5.71 ± 1.27	6.17 ± 1.46	0.004*
Basal FSH (IU/L)	6.71 ± 1.89	6.97 ± 2.52	0.34
AMH (ng/ml)	1.82 ± 0.38	1.75 ± 0.44	0.11
AFC	13.09 ± 6.56	13.39 ± 8.39	0.74
ET or FET; n (%)			0.36
ET	62 (52.1)	104 (46.8)	
FET	57 (47.9)	118 (53.2)	
Protocol of ovarian stimulation or FET; n (%)			0.01*
GnRH-a long protocol	34 (28.6)	43 (19.4)	
Other ovarian stimulation protocol	28 (23.5)	61 (27.5)	
GnRHa-HRT protocol of FET	18 (15.1)	64 (28.8)	
Other protocol of FET	39 (32.8)	54 (24.3)	
Endometrial thickness (mm)	1.01 ± 0.24	0.97 ± 0.23	0.93
Stage of embryo transfer; n (%)			0.42
Cleavage	46 (38.7)	76 (34.2)	
Blastocyst	73 (61.3)	146 (65.8)	
Number of embryo transfer; n (%)			0.02*
1	75 (63.0)	166 (74.8)	
2	44 (37.0)	56 (25.2)	
Pregnancy type; n (%)			<0.01*
No pregnancy	0 (0)	161 (72.5)	
Singleton pregnancy	88 (73.9)	53 (23.9)	
Twin pregnancy	31 (26.1)	8 (3.6)	

Data are expressed as mean ± SD or n (%), unless otherwise stated.

*P < 0.05 was considered statistically significant.

BMI, body mass index; FSH, follicle-stimulating hormone; AMH, anti-Mullerian hormone; AFC, antral follicle count; ET, embryo transfer; FET, frozen embryo transfer; GnRH, gonadotrophin-releasing hormone; HRT, hormone replacement therapy.

### 3.3 Feature selection and MLR model development

MLR analysis identified significant associations between live birth and the following variables: mean initial uterine diameter (odds ratio [OR], 0.72; 95% CI, 0.55-0.94; *P* =0.02), age [OR, 0.92; 95% CI, 0.84–1.01, *P* = 0.08], BMI [odds ratio (OR), 0.89; 95% CI, 0.81–0.98, *P* = 0.02], primary or secondary infertility (OR, 2.03; 95% CI, 0.92–4.57, *P* = 0.08), stage of transferred embryo (OR, 2.23; 95% CI, 0.65–8.19, *P* = 0.21), endometrial thickness(OR, 3.95; 95% CI, 0.92–18.01, *P* = 0.07), number of embryos transferred (OR, 0.57; 95% CI, 0.14-2.24; *P* =0.41), and pregnancy type(OR, 37.95; 95% CI, 17.96-92.974; *P* <0.001) (**Table 3**). A collinearity diagnosis was performed to ensure the exclusion of multicollinearity among predictors. Variance inflation factor (VIF) values for mean initial uterine diameter, age, BMI, infertility type, embryo transfer stage, endometrial thickness, number of embryos transferred, and pregnancy type were all below 10, indicating no severe collinearity between variables (21). The equation describing the probability of the live birth was: *P* = 1/[1+ exp (−X)] where X = 2.1401 − 0.3286 * V1 − 0.0786 * V2 − 0.1147 * V3 + 0.7058 * V4 + 0.0338 * V5 + 1.0904 * V6 + 2.4961 * V7 − 0.5030 * V8, where V1 was the mean initial diameter of the uterus, V2 was the age, V3 was the BMI, V4 was primary or secondary infertility (0 if primary infertility and 1 if secondary infertility), V5 was the stage of the transferred embryo (0 if cleavage and 1 if blastocyst), V6 was the endometrial thickness, V7 was the number of embryo transfers, and V8 was the pregnancy type (1 if singleton pregnancy and 2 if twin pregnancy).

**Table 3:** Odds ratios of the live birth of infertile women with adenomyosis by multivariate analysis of predictor variables.

Predictor variable	OR (95% CI)	*P*-value
Mean initial uterine diameter	0.72 (0.55–0.94)	0.02*
Age	0.92 (0.84–1.01)	0.08
BMI	0.89 (0.81–0.98)	0.02*
Primary or secondary infertility		0.08
Primary infertility	1	
Secondary infertility	2.03 (0.92–4.57)	
Stage of transferred embryo		0.21
Cleavage	1	
Blastocyst	2.23 (0.65–8.19)	
Endometrial thickness	3.95 (0.92–18.01)	0.07
Number of embryo transfer		0.41
1	1	
2	0.57 (0.14–2.24)	
Pregnancy type		<0.001*
Singleton pregnancy	1	
Twin pregnancy	37.95 (17.96–92.974)	

OR, adjusted odds ratio; CI, confidence interval; BMI, body mass index.

*P < 0.05 was considered statistically significant.

### 3.4 Development of the nomogram from the training cohort

**Figure 1:**
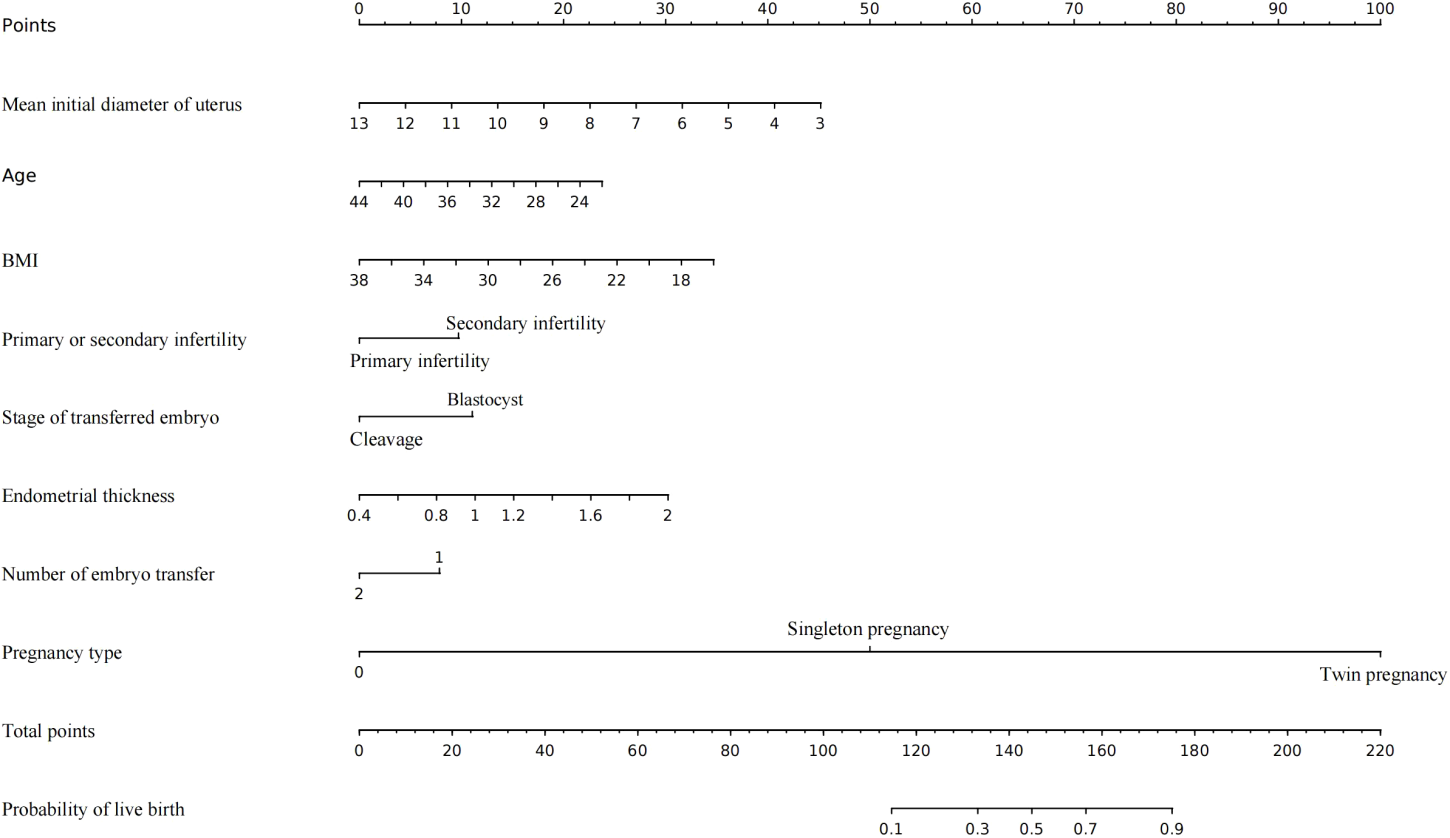
The nomogram to predict the live birth of infertile women with adenomyosis undergoing IVF/ICSI, combining mean initial diameter of uterus, age, BMI, primary or secondary infertility, Stage of embryo transfer, endometrial thickness, number of embryo transfer and pregnancy type. The value of each variable was mapped to a point. The points were summed up and located on the total points line which corresponded to the probability of live birth.

An MLR model integrating clinical expertise was utilized to develop a nomogram for predicting live birth outcomes in infertile women with adenomyosis (**Figure 1**). The nomogram incorporates eight clinically significant predictors: mean initial diameter of uterus, age, BMI, type of infertility, developmental stage of embryo transfer, endometrial thickness, number of embryos transferred, and pregnancy type (**Figure 1**). Each predictor was assigned a weighted point value in the MLR analysis. The total score, derived from summing individual points, was mapped to a predicted probability of live birth on the nomogram’s probability scale (**Figure 1**).

### 3.5 Evaluation of the nomogram

**Figure 2:**
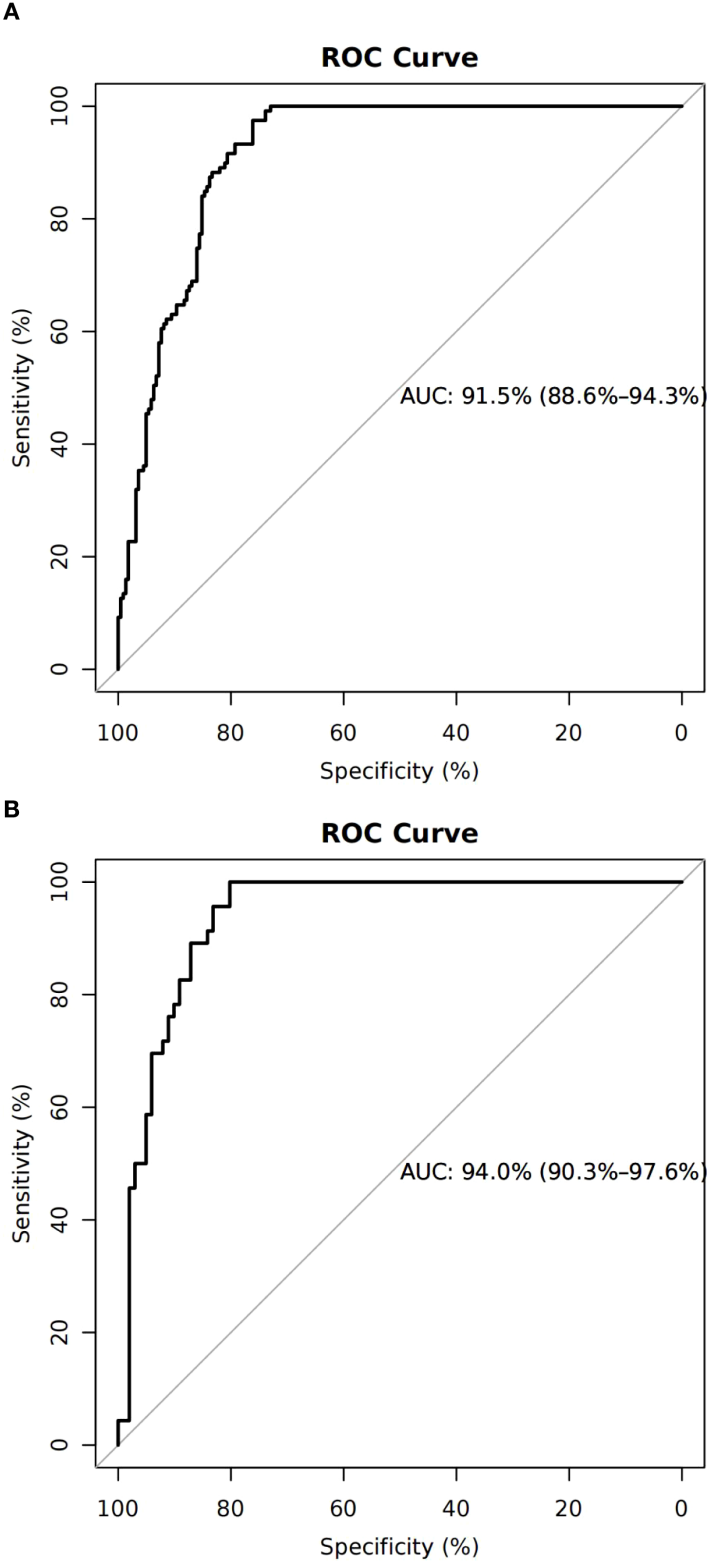
Discrimination and calibration of the training cohort. **(A)** Receiver operating characteristic curve of the model with an area under the curve of 0.915 (95% confidence interval 0.886–0.943). **(B)** Calibration for the training cohort.

The nomogram demonstrated an AUC of 0.915 (95% CI: 0.886–0.943) in the training cohort (**Figure 2A**) and 0.940 (95% CI: 0.903–0.976) in the validation cohort (**Figure 2B**), indicating excellent predictive capability.

**Figure 3:**
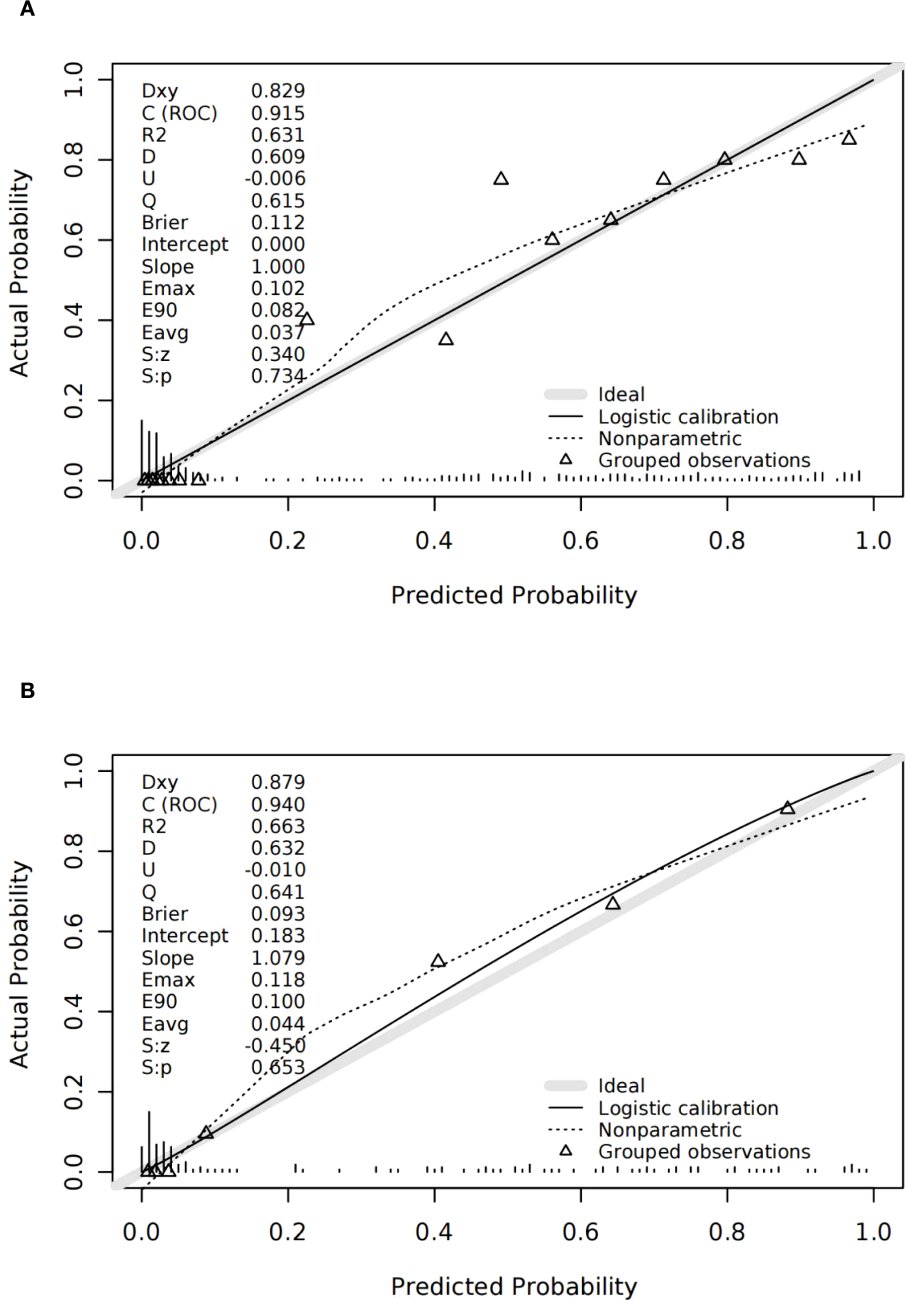
Discrimination and calibration of the validation cohort. **(A)** ROC of the validation cohort; the area under the curve of the nomogram was 0.940 (95% confidence interval 0.903–0.976). **(B)** Calibration for the validation cohort.

Calibration curves confirmed strong consistency between predicted and actual live birth outcomes in both cohorts (**Figures 3A, B**). The H–L test showed no significant miscalibration in the validation cohort.

### 3.6 Clinical utility of the model

**Figure 4:**
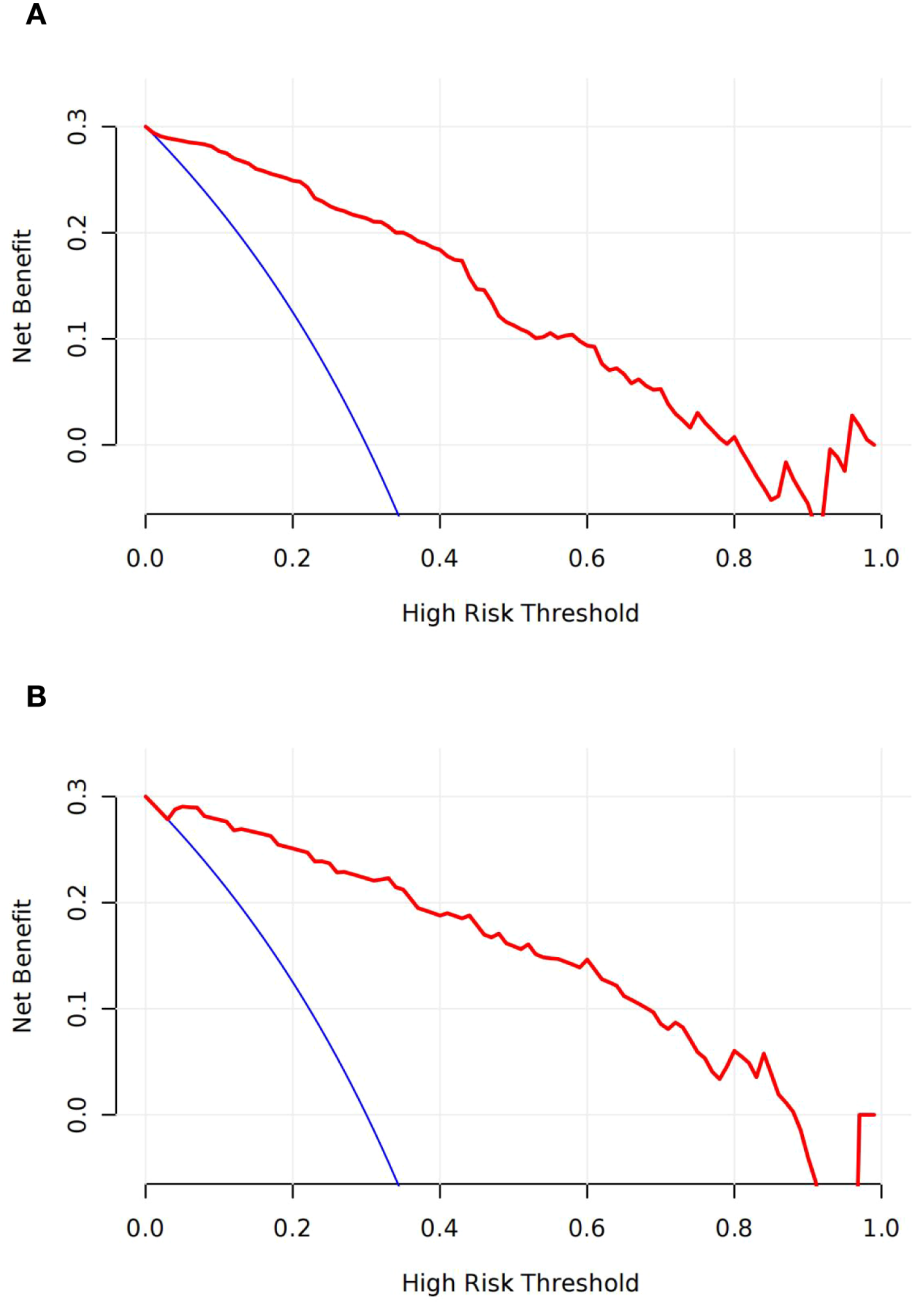
Decision curves analyses (DCA) across all threshold probabilities. The DCA plots the model’s net benefit (y-axis) against threshold probability (x-axis) for predicting the live birth of patients. The DCA of the model in training **(A)** and validation sets **(B)**; the curve has a high net benefit at a low threshold probability of 5%.

Decision curve analysis (DCA) indicated a high net clinical benefit of the model at a threshold probability of 5% (**Figures 4A, B**).

## 4 Discussion

This study presents what is, to our knowledge, the first predictive nomogram developed to estimate live birth rates following IVF/ICSI treatment—including both ET and FET—in infertile women diagnosed with adenomyosis.

Predictive variables for the MLR analysis were selected based on their significance in univariate logistic regression analysis (*P* < 0.1), supported by literature review and clinical expertise. Through backward variable selection, eight significant predictors were identified: mean initial diameter of uterus, age, BMI, type of infertility, developmental stage of embryo transfer, endometrial thickness, number of embryos transferred, and pregnancy type.

Uterine size significantly impacted live birth outcomes in this study. Adenomyosis is characterized by the displacement of endometrial glands and stroma into the myometrium, resulting in uterine enlargement. Previous studies have shown that adenomyosis disrupts the continuity of the uterine junctional zone and alters myometrial architecture, impairs contractility, and affects spiral artery remodeling, thereby compromising embryo implantation. One retrospective study reported that women with adenomyosis and larger uterine volumes exhibited higher miscarriage rates and lower live birth rates (22). Therefore, uterine volume assessment is a key consideration in patients undergoing IVF/ICSI. For women with significantly enlarged uteri, GnRH-a treatment is recommended to reduce lesion size. Our previous research demonstrated that an ultra-long or long ovarian stimulation protocol may be beneficial for fresh embryo transfer in women with adenomyosis (17). BMI also showed a notable influence, with previous research clearly demonstrating that obesity negatively affects live birth rates following IVF. Future research should investigate if lifestyle modifications or bariatric surgery could reverse this adverse effect (23). Age remains a critical prognostic factor in ART, frequently influencing the likelihood of achieving live births or pregnancies (24). Age-related infertility is primarily attributed to diminished ovarian reserve and reduced oocyte/embryo quality due to age-related cellular damage, including increased aneuploidy and decreased mitochondrial activity (25). As adenomyosis incidence rises with age, the average age of participants in this study (approximately 34 years) was associated with adverse pregnancy outcomes. A history of previous pregnancy emerged as a protective factor, indicating that despite adenomyosis, fertility potential and uterine conditions for embryo implantation and development might still be favorable (26). Single blastocyst transfer was associated with higher live birth rates in prior studies. In this study, single blastocyst embryo transfer was recommended due to its higher live birth rate, particularly in women with an enlarged uterus (27, 28). Additionally, adequate endometrial thickness (EMT) is critical for successful pregnancy. A meta-analysis revealed significantly lower pregnancy rates with EMT below 7 mm compared to thicknesses above 7 mm (29). Another study indicated that each millimeter decrease in EMT below 8 mm substantially reduced live birth rates in fresh IVF cycles (30).

The nomogram was constructed using the modeling cohort (n = 341) and validated externally using an independent cohort (n = 147). The predictive model demonstrated high discriminative capability, with an AUC of 0.915 (95% CI: 0.886–0.943) in the training cohort and 0.940 (95% CI: 0.903–0.976) in the validation cohort. Calibration was also robust in both cohorts.

This study has limitations, primarily related to its retrospective nature, which may introduce selection bias. Future prospective randomized controlled trials are required to refine and validate this predictive nomogram further. Despite these limitations, the developed nomogram offers a simple and effective tool for predicting the probability of live birth in women with adenomyosis. Moreover, it provides clinicians with a reliable reference for individual patient consultation and treatment planning.

## 5 Conclusion

In conclusion, this study successfully developed a well-calibrated nomogram to predict the live birth rate in infertile women with adenomyosis undergoing IVF/ICSI treatment. The nomogram incorporates eight clinically significant predictors: mean initial diameter of uterus, age, BMI, type of infertility, developmental stage of embryo transfer, endometrial thickness, number of embryos transferred, and pregnancy type. The nomogram provides clinicians with a valuable tool for enhancing clinical decision-making and increasing the probability of achieving live births in this patient population.

## Data Availability

The raw data supporting the conclusions of this article will be made available by the authors, without undue reservation.

## References

[1] Upson K Missmer SA. Epidemiology of adenomyosis. Semin Reprod Med. (2020) 38:89–107. doi: 10.1055/s-0040-1718920, PMID: , PMID: 33105509 PMC7927213

[2] Li Y-W Liu Y-T Wang S Shi H-H Fan Q-B Zhu L. Clinical manifestations of adenomyosis patients with or without coexisting endometriosis. Chin Med J. (2018) 131:2495. doi: 10.4103/0366-6999.243572, PMID: , PMID: 30334538 PMC6202587

[3] Guo S Zhang D Lu X Zhang Q Gu R Sun B. Hypoxia and its possible relationship with endometrial receptivity in adenomyosis: a preliminary study. Reprod Biol Endocrinol. (2021) 19:7. doi: 10.1186/s12958-020-00692-y, PMID: , PMID: 33419445 PMC7791798

[4] Bulun SE Yildiz S Adli M Wei J-J. Adenomyosis pathogenesis: insights from next-generation sequencing. Hum Reprod Update. (2021) 27:1086–97. doi: 10.1093/humupd/dmab017, PMID: , PMID: 34131719 PMC8543024

[5] Levy G Dehaene A Laurent N Lernout M Collinet P Lucot J-P. An update on adenomyosis. Diagn Interventional Imaging. (2013) 94:3–25. doi: 10.1016/j.diii.2012.10.012, PMID: , PMID: 23246186

[6] Moawad G Kheil MH Ayoubi JM Klebanoff JS Rahman S Sharara FI. Adenomyosis and infertility. J Assist Reprod Genet. (2022) 39:1027–31. doi: 10.1007/s10815-022-02476-2, PMID: , PMID: 35347501 PMC9107544

[7] Shinohara S Okuda Y Hirata S Suzuki K. Adenomyosis as a potential risk factor for adverse pregnancy outcomes: A multicenter case-control study. Tohoku J Exp Med. (2020) 251:231–9. doi: 10.1620/tjem.251.231, PMID: , PMID: 32684535

[8] Vigano P Corti L Berlanda N. Beyond infertility: obstetrical and postpartum complications associated with endometriosis and adenomyosis. Fertility Sterility. (2015) 104:802–12. doi: 10.1016/j.fertnstert.2015.08.030, PMID: , PMID: 26348274

[9] Vercellini P Viganò P Bandini V Buggio L Berlanda N Somigliana E. Association of endometriosis and adenomyosis with pregnancy and infertility. Fertility Sterility. (2023) 119:727–40. doi: 10.1016/j.fertnstert.2023.03.018, PMID: , PMID: 36948440

[10] Younes G Tulandi T. Effects of adenomyosis on *in vitro* fertilization treatment outcomes: a meta-analysis. Fertility Sterility. (2017) 108:483–490.e3. doi: 10.1016/j.fertnstert.2017.06.025, PMID: , PMID: 28865548

[11] Kim MS Jang JH Park S Ahn EH Jung SH. Effect of adenomyosis on adverse obstetrical outcomes in twin pregnancies achieved with assisted reproductive technology. J Obstetrics Gynaecology. (2021) 41:1225–9. doi: 10.1080/01443615.2020.1867969, PMID: , PMID: 33890530

[12] Szubert M Koziróg E Olszak O Krygier-Kurz K Kazmierczak J Wilczynski J. Adenomyosis and infertility-review of medical and surgical approaches. Int J Environ Res Public Health. (2021) 18:1235. doi: 10.3390/ijerph18031235, PMID: , PMID: 33573117 PMC7908401

[13] Cozzolino M Tartaglia S Pellegrini L Troiano G Rizzo G Petraglia F. The effect of uterine adenomyosis on IVF outcomes: a systematic review and meta-analysis. Reprod Sci. (2022) 29:3177–93. doi: 10.1007/s43032-021-00818-6, PMID: , PMID: 34981458

[14] Wu Y Yang R Lin H Cao C Jiao X Zhang Q. A validated model for individualized prediction of live birth in patients with adenomyosis undergoing frozen–thawed embryo transfer. Front Endocrinol. (2022) 13:902083. doi: 10.3389/fendo.2022.902083, PMID: , PMID: 35685210 PMC9171040

[15] Bazot M Daraï E. Role of transvaginal sonography and magnetic resonance imaging in the diagnosis of uterine adenomyosis. Fertility Sterility. (2018) 109:389–97. doi: 10.1016/j.fertnstert.2018.01.024, PMID: , PMID: 29566851

[16] Van den Bosch T Dueholm M Leone FPG Valentin L Rasmussen CK Votino A. Terms, definitions and measurements to describe sonographic features of myometrium and uterine masses: a consensus opinion from the Morphological Uterus Sonographic Assessment (MUSA) group. Ultrasound Obstetrics Gynecology. (2015) 46:284–98. doi: 10.1002/uog.14806, PMID: , PMID: 25652685

[17] Ge L Li Y Guan S Cui L Chen Z-J. Effects of ovarian stimulation protocols on outcomes of assisted reproductive technology in adenomyosis women: a retrospective cohort study. Front Endocrinol. (2023) 14:1198779. doi: 10.3389/fendo.2023.1198779, PMID: , PMID: 37664864 PMC10472936

[18] Puissant F Van Rysselberge M Barlow P Deweze J Leroy F. Embryo scoring as a prognostic tool in IVF treatment. Hum Reprod. (1987) 2:705–8. doi: 10.1093/oxfordjournals.humrep.a136618, PMID: , PMID: 3437050

[19] Man Y Bian Y Zhao S Zhao R Xu X Wei D. The effect of different endometrial preparations on women with polycystic ovary syndrome undergoing initial frozen embryo transfer: A historical cohort analysis. Acta Obstetricia Gynecologica Scandinavica. (2021) 100:1116–23. doi: 10.1111/aogs.14058, PMID: , PMID: 33616957

[20] Huang Y Liang C He L Tian J Liang C Chen X. Development and validation of a radiomics nomogram for preoperative prediction of lymph node metastasis in colorectal cancer. JCO. (2016) 34:2157–64. doi: 10.1200/JCO.2015.65.9128, PMID: , PMID: 27138577

[21] Fox J Monette G. Generalized collinearity diagnostics. J Am Stat Assoc. (1992) 87:178–83. doi: 10.1080/01621459.1992.10475190

[22] Li X Pan N Zhang W Wang Y Ge Y Wei H. Association between uterine volume and pregnancy outcomes in adenomyosis patients undergoing frozen-thawed embryo transfer. Reprod BioMedicine Online. (2021) 42:384–9. doi: 10.1016/j.rbmo.2020.10.002, PMID: , PMID: 33243661

[23] Sermondade N Huberlant S Bourhis-Lefebvre V Arbo E Gallot V Colombani M. Female obesity is negatively associated with live birth rate following IVF: a systematic review and meta-analysis. Hum Reprod Update. (2019) 25:439–51. doi: 10.1093/humupd/dmz011, PMID: , PMID: 30941397

[24] Loy SL Cheung YB Fortier MV Ong CL Tan HH Nadarajah S. Age-related nomograms for antral follicle count and anti-Mullerian hormone for subfertile Chinese women in Singapore. PloS One. (2017) 12:e0189830. doi: 10.1371/journal.pone.0189830, PMID: , PMID: 29240820 PMC5730199

[25] Cimadomo D Fabozzi G Vaiarelli A Ubaldi N Ubaldi FM Rienzi L. Impact of maternal age on oocyte and embryo competence. Front Endocrinol (Lausanne). (2018) 9:327. doi: 10.3389/fendo.2018.00327, PMID: , PMID: 30008696 PMC6033961

[26] Wang Y Hu Y Jiang P Kong W Gong C Chen Y. Establishment and validation of a nomogram model for predicting adverse pregnancy outcomes of pregnant women with adenomyosis. Arch Gynecol Obstet. (2024) 309:2575–84. doi: 10.1007/s00404-023-07136-z, PMID: , PMID: 37490056

[27] Holden EC Kashani BN Morelli SS Alderson D Jindal SK Ohman-Strickland PA. Improved outcomes after blastocyst-stage frozen-thawed embryo transfers compared with cleavage stage: a Society for Assisted Reproductive Technologies Clinical Outcomes Reporting System study. Fertility Sterility. (2018) 110:89–94.e2. doi: 10.1016/j.fertnstert.2018.03.033, PMID: , PMID: 29908769

[28] Glujovsky D Farquhar C Retamar AMQ Sedo CRA Blake D. Cleavage stage versus blastocyst stage embryo transfer in assisted reproductive technology. Cochrane Database Systematic Rev. (2016) 30(6):CD002118. doi: 10.1002/14651858.CD002118.pub5, PMID: , PMID: 27357126

[29] Kasius A Smit JG Torrance HL Eijkemans MJC Mol BW Opmeer BC. Endometrial thickness and pregnancy rates after IVF: a systematic review and meta-analysis. Hum Reprod Update. (2014) 20:530–41. doi: 10.1093/humupd/dmu011, PMID: , PMID: 24664156

[30] Liu KE Hartman M Hartman A Luo Z-C Mahutte N. The impact of a thin endometrial lining on fresh and frozen-thaw IVF outcomes: an analysis of over 40–000 embryo transfers. Hum Reprod. (2018) 33:1883–8. doi: 10.1093/humrep/dey281, PMID: , PMID: 30239738 PMC6145412

